# Starting well to stay well - randomised controlled trial of Whitu, an app for improving the well-being of university students

**DOI:** 10.1192/j.eurpsy.2024.1144

**Published:** 2024-08-27

**Authors:** H. Thabrew

**Affiliations:** Psychological Medicine, University of Auckland, Auckland, New Zealand

## Abstract

**Introduction:**

University students often face challenges to their well-being and up to a third develop mental health problems. Given high rates of smartphone use among this group, app-based digital mental health interventions may play a role in preventing these problems. Previously demonstrated to improve well-being and mental health outcomes in young people aged 16-25, ‘Whitu: seven ways in seven days’ is a well-being app based on positive psychology, cognitive behaviour therapy (CBT) and psychoeducation principles.

**Objectives:**

This randomised controlled trial was undertaken to evaluate the efficacy, usability and acceptability of Whitu with first year university students.

**Methods:**

Ninety first year university students were recruited via a social media advertising campaign to take part in a prospective randomised controlled trial of Whitu against a standard university self-help website, with 45 participants in each arm. Primary outcomes were changes in well-being on the World Health Organisation 5-item well-being index (WHO-5) and short Warwick-Edinburgh mental well-being escale (SWEMWBS). Secondary outcomes were changes in depression on the Centre for Epidemiological Studies Depression Scale (CES-D), anxiety on the Generalised Anxiety Disorder seven item scale (GAD-7), self-compassion on the Self Compassion Scale- Short Form (SCS-SF), stress on the 10-item Perceived Stress Scale (PSS-10), sleep on the single-item Sleep Quality Scale (SQS), and self-reported acceptability of the app. Outcomes were evaluated at baseline, four weeks (primary study endpoint) and three months.

**Results:**

At 4 weeks, participants in the intervention group experienced significantly higher mental well-being and significantly lower depression compared to controls. Emotional well-being among the Whitu group was greater in the intervention group at 3 months. Other outcomes did not differ between groups. User feedback was positive, with 88% of those who provided feedback saying they would recommend the app to a friend.

**Conclusions:**

Our findings provide preliminary evidence that Whitu is an acceptable and more effective, scalable and multi-modal means of improving some aspects of well-being and mental health among university students than direction to a self-help website.

**Disclosure of Interest:**

None Declared

